# 

**Published:** 1887-10-15

**Authors:** 


CONTENTS.

**Science and Socialism** - - - - - - 35

**Words of Consolation.**—Chapter LIV. The Sufferings of Christ - - - - - - - - - - 36

**Healing the Sick.** By the Right Rev. the LORD BISHOP OF LONDON - - - - - - - - - 37

**Sick Soldiers and their Nurses** - - - - 37

**Cruel Treatment of a Lunatic by her Parents** - 38

**A National Pension Fund for Hospital Nurses and Officials.** By HENRY C. BURDETT - - - - 39

**Professional Notes.** By a PHYSICIAN - - - 41

**Notes and News.**—Miscellaneous Items.—The Hospitals Association Meetings—Sir Andrew Clark and the London Hospital.—Mahometanism and Christianity.—Vacancies.—A Football Match.—Experimental Laboratories.—West of Scotland Seaside Homes.—Smoking.—The Scarlet Fever Epidemic.—The Grimsby and District Hospital.—The Great Northern Central Hospital.—"The Golden Hand."—The Royal Portsmouth Hospital.—Miss Twining on Village Life.—Robbing a Hospital Collecting Cage.—The *Truth* Christmas Toy Fund.—Deputation of Unemployed to the Local Government Board 42

**Annotations.**—New Wars: New Weapons.—The Digestibility of Food.—Butter or Butterine - - - - - - 45

**Small-pox Specifics—Is Vaccination a Failure** - 46

**East and West.** By LESLIE KEITH.—Chapter III. Lavender Lane - - - - - - - - - - 47

**A Nurse's View of Hospital Patients** - - - 49

**Old Linen and Calico** - - - - - - 49

**The Keighley Cottage Hospital** - - - - 49

**Amusements with Prizes, for all Ages.**—For Men and Women.—For Convalescents.—The Children's Corner - 50

**Hospitals, Doctors, Treatment, and Attendance.** The In- and Out-patients' Hospital Diary - - - - 51

